# Depleting Cellular Retinoic Acid Binding Protein 1 Impairs UPR^mt^

**DOI:** 10.33696/signaling.4.102

**Published:** 2023

**Authors:** Chin-Wen Wei, Thomas Lerdall, Fatimah Najjar, Li-Na Wei

**Affiliations:** 1 Department of Pharmacology, University of Minnesota, Minneapolis, MN 55455, USA

**Keywords:** Reactive oxygen species, Unfolded protein response, Crabp1, eIF2α

## Abstract

Mitochondrial dysfunction underlines neurodegenerative diseases which are mostly characterized by progressive degeneration of neurons. We previously reported that *Cellular retinoic acid Binding protein 1* (*Crabp1*) knockout (CKO) mice spontaneously developed age-dependent motor degeneration, with defects accumulated in spinal motor neurons (MNs), the only cell type in spinal cord that expresses CRABP1. Here we uncovered that mitochondrial DNA (mtDNA) content and the expression of genes involved in respiration were significantly reduced in CKO mouse spinal cord, accompanied by significantly elevated reactive oxygen species (ROS) and unfolded protein load, indicating that CRABP1 deficiency caused mitochondrial dysfunction. Further analyses of spinal cord tissues revealed significant reduction in the expression and activity of superoxide dismutase 2 (SOD2), as well as defected mitochondrial unfolded protein response (UPR^mt^) pathway, specifically an increase in ATF5 mRNA but not its protein level, which suggested failure in the translational response of ATF5 in CKO. Consistently, eukaryotic initiation factor-2α, (eIF2α) phosphorylation was reduced in CKO spinal cord. In a CRABP1 knockdown MN1 model, siCrabp1-MN1, we validated the cell-autonomous function of CRABP1 in modulating the execution of UPR^mt^. This study reveals a new functional role for CRABP1 in the execution of mitochondrial stress response, that CRABP1 modulates eIF2α phosphorylation thereby contributing to ATF5 translational response that is needed to mitigate mitochondria stress.

## Introduction

Cellular Retinoic Acid Binding Protein 1 (CRABP1) is a high affinity, cytosolic binding protein for retinoic acid (RA) [1]. Recent studies have established CRABP1’s functional role as a specific cytosolic RA signal mediator that acts to modulate two cytosolic signaling pathways, the extracellular signal-regulated kinases/mitogen-activated protein kinases (ERK/MAPK) and calcium-calmodulin-dependent protein kinase II (CaMKII) pathways [2]. These recently uncovered CRABP1-mediated cytosolic activities are referred to as non-canonical activities of RA, in contrast to widely known canonical activities of RA mediated by nuclear RA receptors (RARs) that typically act to regulate gene expression [3,4]. Using *Crabp1* gene knockout (CKO) mice and cell models, we have demonstrated that CRABP1 plays roles in multiple cellular processes and disease conditions [5], such as neuron stem cell proliferation [6], motor neuron (MN) function [7], endocrine homeostasis [8,9], exosome secretion [10] and adipocyte hypertrophy [11,12]. Most of these processes involve the modulation of either the ERK or the CaMKII pathway by CRABP1. Our most recent data demonstrated that CRABP1 is vital to the health of spinal MNs and motor activities, specifically in maintaining neuromuscular junctions (NMJs) [7].

We have previously found that CRABP1 is highly expressed in spinal cord, specifically in spinal MNs, not in glia cells [7] and reported that CKO mice, that lost CRABP1, spontaneously develop age-dependent, amyotrophic lateral sclerosis (ALS)-like motor degenerative disease. Others’ studies have also shown that Crabp1 expression is reduced in MN disease models, such as spinal muscular atrophy and amyotrophic lateral sclerosis (ALS) [13–15]. The phenotype of CKO mice is initiated from weakened grip strength and abnormal NMJ at two-month-old, followed by decreased spinal MN numbers and the gray matter area of lumbar spinal cord in aged CKO mice [7]. It is known that mitochondrial dysfunction underlines numerous diseases including cardiovascular diseases, metabolic disorders, and neurodegenerative diseases, such as ALS. Interestingly, in CKO embryonic stem cells (unpublished) and adipocytes [11], there is a significant reduction in mitochondrial DNA (mtDNA) content. Additionally, our unpublished preliminary studies have detected increased abnormality in mitochondrial function in CKO spinal cord tissues. This current study aims to understand how CRABP1 deficiency affects mitochondria function, especially in spinal cord where MN is the cell type highly expressing CRABP1. The loss of CRABP1 in spinal MNs may contribute to the ALS-like motor-degenerative phenotype of CKO mice.

Among many causes of mitochondrial dysfunction, stress appears to be one of common triggers that can induce mitochondrial DNA (mtDNA) mutations, impair mitochondrial respiration, and alter mitochondrial membrane potential (MMP) and Ca^2+^ permeability [16–18]. The most common mitochondrial stressors are reactive oxygen species (ROS) and the accumulation of unfolded proteins. The common defense mechanism to eliminate these mitochondrial stressors is the activation of mitochondrial unfolded protein response (UPR^mt^) and antioxidant pathways that elevate enzymatic antioxidants including superoxide dismutase (SOD), glutathione peroxidase (Gpx), peroxiredoxins (Prx), and catalase, as well as certain nonenzymatic antioxidants such as vitamin C and glutathione (GSH) [19]. When damage persists or increases, severely damaged mitochondria will be removed via mitophagy processes. However, if all these mechanisms fail, the cell will undergo apoptosis and be eliminated [20].

Mitochondria contain more than 1000 proteins, most of these proteins are encoded by nuclear genes whose protein products are imported into mitochondrial matrix [21]. Under mitochondrial stresses, UPR^mt^ is initiated by phosphorylating a specific translation initiation factor, eukaryotic initiation factor-2α (eIF2α). The phosphorylated eIF2α causes global repression of protein synthesis while activating the translation of UPR^mt^ transcription factors, such as ATF4, ATF5, and CHOP, to elevate their protein levels in order to enhance the expression of genes coding for antioxidant enzymes [22,23] and UPR^mt^ proteins such as mitochondrial chaperone proteins and proteases [24].

In the CKO mouse spinal tissue where MNs were severely damaged, we detected altered mitochondrial content in preliminary examination. This prompted us to examine how CRABP1 may play a role in mitochondrial homeostasis or metabolism to affect cellular health. The goal in this study is to first identify potential events/pathways in maintaining mitochondrial or metabolic homeostasis that may be modulated by CRABP1. This may provide new insight into MN defects that lead to the ALS-like motor degenerative phenotype of CKO mice.

## Materials and Methods

### Animals

Three to four months-old mice were used for the experiments. Mice were maintained in the University of Minnesota animal facility on a 14-hour light/10-hour dark cycle (lights on/off at 0600/2000) at 22 ± 1°C with ad-libitum food (Inotiv, #2918 Teklad Global 18% protein Rodent Diet) and water. C57BL/6J background CKO mice were generated as described [12,25]. Briefly, CKO mice were generated from a *Crabp1*-targeted DE3 (ES) clone containing a 5bp Not1 insertion in exon 1 (the fifth codon of the *Crabp1* coding region) to ablate *Crabp1* expression. The CKO ES clone was injected to generate CKO chimeric mice in the University of Minnesota transgenic facility. These mice were subsequently backcrossed onto a C57BL/6J (#000664, Jackson Laboratory) background for 12 generations to establish C57BL/6J background CKO mice maintained in house. Heterozygous *Crabp1*^+/−^ mice were bred with each other to generate homozygous wild-type (WT) and CKO homozygous mice. All experimental procedures were conducted following the NIH guidelines and the protocols approved by the University of Minnesota Institutional Animal Care and Use Committee.

### Spinal cord tissue isolation

Mice were euthanized by CO_2_ asphyxiation. Immediately after, the mice were processed to isolate the spinal cord. The lumbar region of the spinal cord was flushed out with a butterfly needle using a PBS-filled 10 ml syringe. The spinal tissues were digested with the Neural Tissue Dissociation Kits (MACS #130-092-628) according to the manufacturer’s instructions. Cell suspensions were then subjected to Percoll gradients (Cytiva #17-5445-02) to remove debris [26]. The cell suspensions were adjusted to 7 ml with HBSS, and 3 ml of stock isotonic Percoll (SIP) solution was added to make a final 30% SIP. The cells containing 30% Percoll were carefully transferred onto the top of the 70% Percoll layer. The mixture was then centrifuged at 650g at room temperature for 25 minutes (with the braking level set to 0). The layer of debris was gently removed from the top and washed with FACS buffer, and single-cell suspensions were used for flow cytometry.

### MNs isolation

MNs from mouse spinal cord were isolated as described [27]. Briefly, the spinal tissues were digested with the Neural Tissue Dissociation Kits (MACS #130-092-628) according to the manufacturer’s instructions. Cell suspensions were adjusted to 4 ml NABG medium, a neuralbasal medium (Gibco #21103049) containing 2% B27 (Gibco #17504044) and 2 mM glutamine. The cell content was carefully transferred onto the top of a 4 ml separation solution, 87.6% DMEM/F12 medium, 12.4% OptiPrep (Sigma-aldrich #D1556). The mixture was then centrifuged at 800g at 4°C for 15 minutes (with the braking level set to 0). The mixture was separated into three layers: F1, F2, and F3. MNs were isolated from the F2 layer and washed with NABG medium. The pellet containing MNs was processed for mtDNA quantification.

### Mitochondrial DNA quantification

Cell total DNA was extracted using a genomic DNA extraction kit (Qiagen Dneasy blood & tissue kit) according to the manufacturer’s instructions. COII expression for mitochondrial DNA abundance analysis by SYBR^™^ Green PCR Master Mix (ThermoFisher, # K0253) with 50 ng of total DNA in combination of COII.F, 5’ TGAGCCATCCCTTCACTAGG 3’; COII.R, 5’ TGAGCCGCAAATTTCAGAG 3’ or β-actin F, 5’ TGTTCCCTTCCACAGGGTGT 3’; β-actin R, 5’ TCCCAGTTGGTAACAATGCCA 3’ primers [28] . Real-time RT-PCR was conducted on Mx3000P QPCR Systems (Agilent). Relative mtDNA were measured and calculated by normalizing β-*actin* expression level to *COII* level.

### Quantitative RT-PCR

RNA was extracted by TRIzol Reagent (Ambion). RNA concentration was measured with the NanoDrop and cDNA was synthesized by High-Capacity cDNA Reverse Transcription Kit (Applied Biosystems^™^ #4368814). Quantitative RT-PCR was performed using the SYBR^™^ Green PCR Master Mix (ThermoFisher, # K0253). Real-time RT-PCR was conducted on Mx3000P QPCR Systems (Agilent). Primers listed in Table S1.

### Flow cytometry

To examine mitochondrial membrane potential (MMP), cells were incubated with 0.1 μM MitoTracker Deep Red at 37°C for 30 minutes in DMEM without serum. For ROS level detection, cells were incubated in PBS with 2.5 μM CellROX or MitoSOX Red at 37°C for 30 minutes. To measure the unfolded protein load, we used a thiol probe to label the cells [29]. Cells were stained with 50 μM Tetraphenylethene maleimide (TPE-MI) (MCE # HY-143218) in PBS at 37°C for 30 minutes in PBS. Each stock solution was diluted with pre-warmed (37°C) PBS or growth medium without serum to a final working concentration. After incubation, live/dead cells staining was performed with zombie dye (Biolegend) in PBS on ice for 20 minutes or resuspended in FACS buffer with propidium iodide (Sigma-Aldrich P4684). Flow cytometry experiments were performed on BD LSR II (BD Biosciences) and analyzed using FlowJo software.

### Intracellular ATP detection

Intracellular ATP was determined using luciferase probes that measure ATP. 0.015 g of tissues was homogenized in 500 μl of cold lysis buffer (25mM Tris/phosphate, 4 mM EGTA, 2 mM DTT, 1% Triton X-100, and 10% glycerol, pH 7.8). The homogenate was then centrifuged at 13,000g at 4°C for 5 minutes, and the supernatant was collected for the assay. Luciferase assay was performed with Luminescent ATP Detection Assay Kit (Abcam #ab113849) according to the manufacturer’s instructions.

### SOD activity

0.02 g of tissues were homogenized in 100 μl of cold 20 mM HEPES buffer, 1 mM EGTA, 210 mM mannitol, and 70 mM sucrose, pH 7.2. The homogenate was then centrifuged at 1,500g at 4°C for 5 minutes, and the supernatant was collected for the assay. For serum isolation, whole blood was collected via cardiac puncture and allowed to coagulate for 30 minutes at room temperature. The Samples were then centrifuged at 10,000g at 4°C for 20 minutes, and the supernatants were collected. SOD activity was measured with Superoxide Dismutase Assay Kit (Cayman # 706002) following the manufacturer’s instructions.

### Western blotting

Cells were lysed in lysis buffer (50 mM HEPES, 0.1 mM EGTA, 0.1 mM EDTA, 120 mM NaCl, 0.5% sodium deoxycholate, 0.1% SDS, 1 mM NaF, 1 mg/ml NaVO4, 1 mM PMSF and 1X Protease Inhibitor Cocktail (ThermoFisher #78439)) and incubated on ice for 20 mins and then centrifuged at 14000g, 4°C for 20 min. Protein quantification of whole cell lysate supernatant was performed using Bradford Assay (Biorad #500–0006). Protein lysates were boiled in 4X sample buffer (Bio-Rad #1610747) for 10 min and electrophoresed on SDS polyacrylamide gels and transferred onto PVDF membranes (Millipore #IPVH00010). The membrane was then blocked with 5% non-fat milk in 1x TBST at room temperature for 1 hr. Membranes were washed four times for 5 min with 1x TBST (0.1% Tween-20 in TBS) and incubated with primary antibodies: ATF5 (Abcam #EPR18286), α-tubulin (Santa Cruz #sc-5286), Phospho-eIF2α (Cell Signaling #9721), eIF2α ( Santa Cruz #sc-11386), and GAPDH (Cell Signaling #5174) with antibody buffer (0.1% Tween 20 and 2% BSA in TBS) overnight and then in secondary horseradish peroxidase-conjugated antibody (HRP; GeneTex). HRP signal was detected using Immobilon Western Chemiluminescent HRP substrate (Advansta #K-12045-d50). Images were acquired with Bio-Rad ChemiDoc Imager (Bio-Rad Laboratories) and the relative level of protein was analyzed by ImageLab software.

### Stable cell line generation

HEK-293T and MN1 cell lines were cultured in complete DMEM medium (Gibco #11965) containing 100 U/mL penicillin, 100 mg/mL streptomycin, and 10 % heat-inactivated FBS. Cells were regularly tested for mycoplasma contamination. The pGIPZ-siCrabp1(V3LHS_358805) and pGIPZ-non-silencing negative control plasmids were purchased from UMGC RNAi (RNA Interference of University of Minnesota Genomics Center). All plasmid DNA were purified using the PureLink HiPure Plasmid Filter Midiprep Kit (Invitrogen #K210014).

For lentivirus production, 2×10^6^ HEK-293T cells were seeded in complete DMEM medium without antibiotics in 10 cm dish overnight. 9.6 μg of target plasmid, 7.2 μg of psPAX2 packaging plasmid, 2.4 μg of pMD2.G envelope plasmid was cotransfected into cells with Lipofectamine 2000 transfection reagent (Invitrogen) following the manufacturer’s protocol. The media was changed to 6 ml of fresh complete DMEM medium containing 1% BSA after 6 hours. Infectious lentiviruses were harvested at 24- and 48-hours post-transfection and filtered through 0.45 μM pore cellulose acetate filters.

For transduction, 1×10^5^ MN1 cells were seeded in complete DMEM medium in 6-well plate overnight. 2 ml of lentivirus with 8 μg/ml polybrene (Millpore TR-1003-G) were added to the cells, and the cells were spun at 800g, 37°C for 60 minutes. The Lentivirus was removed, and fresh medium was added after 24 hours, and puromycin selection was started at 48 hours post-transfection. Cells were selected and maintained in the same medium as described above with the addition of 3 μg/ml puromycin. After puromycin selection, stable MN1 cells were collected and *Crabp1* expression was examined by qPCR.

### Statistical analysis

Sample size chosen for *in vivo* experiments: Flow cytometry analysis, n=4–6 mice per group. SOD activity, n=4–8 mice per group. No animals were excluded from the analyses. Two-tailed Student’s t-test was used when appropriate for comparison among the groups. Data were normally distributed, and variance was similar among groups that were being statistically compared. Data were presented as means ± SD. The comparison was considered statistically significant when p values ≤ 0.05 (* p < 0.05; * * p < 0.01; * * * p < 0.001). Prism 6.0 (GraphPad) was used for plotting data and statistical analysis.

## Result

### Mitochondrial content and metabolism are impaired in CKO’s spinal cord

**Figure 1.**
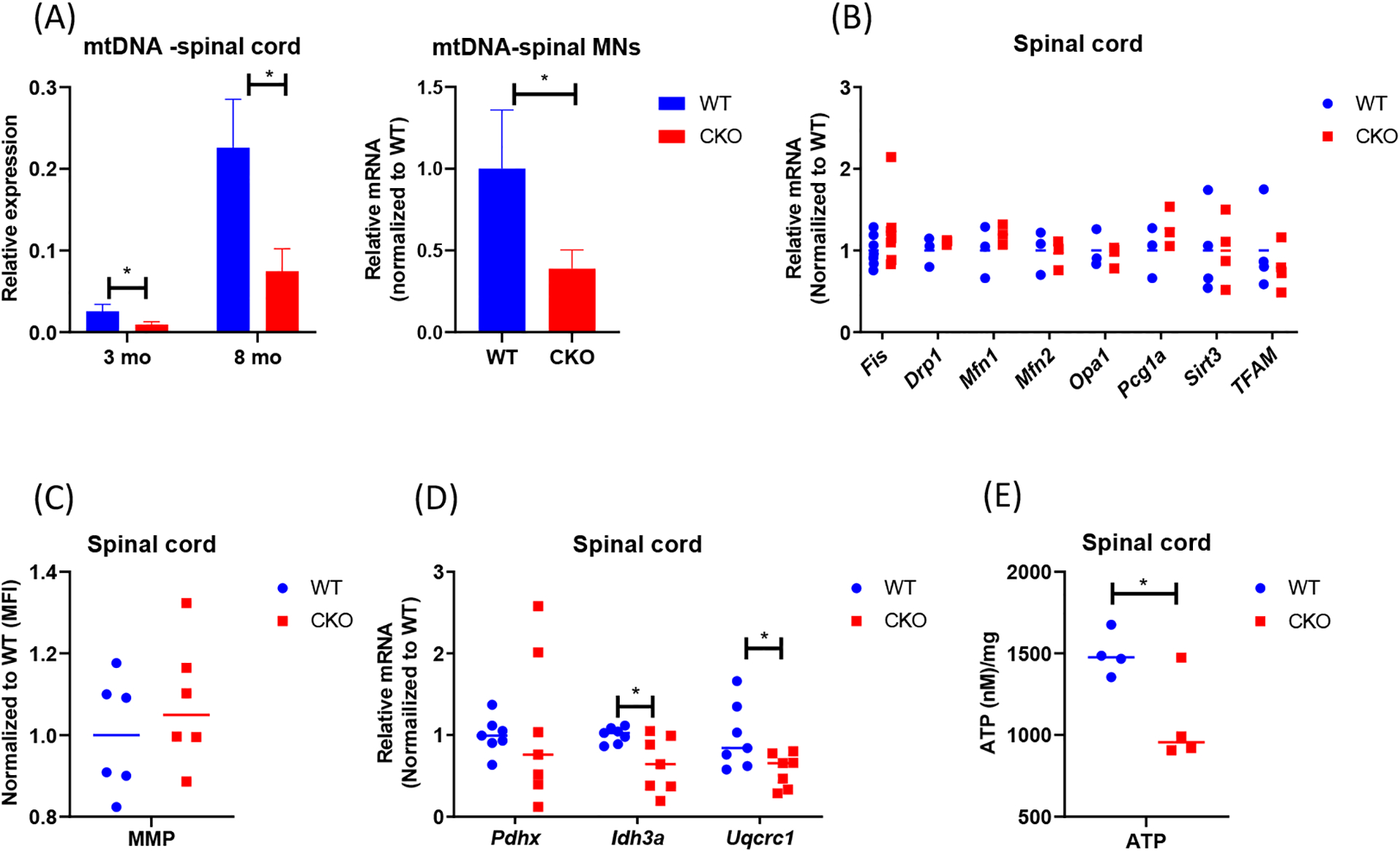
Mitochondrial content and metabolism are impaired in CKO spinal cord. **(A)** qPCR to determine mtDNA contents. mtDNA was determined by monitoring *COll* expression and normalized to β-*actin*. CKO’s spinal MNs value was normalized to that of WT. n=3. Error bars show means ± SD. **(B)** qPCR to determine the expression of genes for mitochondrial biogenesis in spinal cord tissues. *GAPDH* or *RPL19* was used as an internal control, and data were normalized to WT. n=3–7 **(C)** Mitochondrial membrane potential (MMP). Cells from spinal cord tissues were incubated with 0.1 μM MitoTracker Deep Red at 37°C for 30 min and analyzed by flow cytometry. Cells were gated based on single and live cells (propidium iodide^−^). Results were normalized to WT. The n=6, pooled results were from three independent experiments. MFI = Mean fluorescence intensity. **(D)** qPCR to determine the expression of mitochondrial metabolic genes in spinal cord tissues. Normalized to *RPL19* as an internal control, and data were normalized to WT. n=7. (E) ATP levels were detected by luminescent. n=4. Student’s t-test, *p<0.05.

To determine if mitochondrial function or its regulation was impaired in adult CKO spinal cord, we first compared mtDNA content of spinal cord tissues between adult WT and CKO mice. As shown in Figure 1A, mtDNA content was significantly lower in CKO’s spinal cord, as well as spinal MNs, at both 3-month-old and 8-month-old stages. Also, the expression level of mtDNA-encoded cytochrome c oxidase subunits 1 (MT-CO1) was lower in CKO’s spinal cord (Figure S1), revealing mitochondrial defects in adult CKO’s spinal cord. We then examined the expression of genes related to mitochondrial biogenesis. Surprisingly, there was no significant difference between WT and CKO’s tissues in the expression of key mitochondrial biogenesis genes such as *Fis, Drp1, Mfn1, Mfn2, Opa1, Pgc1a, Stirt3*, and *TFAM* (Figure 1B). This result suggested that decrease in mtDNA content of CKO spinal cord was not due to altered mitochondrial biogenesis. We next stained the spinal cord with MitoTracker Deep Red, a dye monitoring MMP, because depolarization of MMP can also be associated with mitochondria dysfunction. The result showed no difference in the MMP of spinal cord between WT and CKO mice (Figure 1C). However, in analyzing the expression of genes related to respiration, we detected significantly reduced expression of *Idh3a*, an enzyme responsible for generating α-ketoglutarate from isocitrate in the TCA cycle, as well as *Uqcrc1*, a subunit of complex III in the mitochondrial respiratory chain, in CKO as compared to WT mice (Figure 1D). We further measured intracellular ATP in spinal cord tissue between WT and CKO mice and found significantly decreased intracellular ATP content in CKO’s spinal cord (Figure 1E). Together, these results indicate impaired mitochondrial function, but not biogenesis, in CKO’s spinal cord. The functional deterioration of mitochondria may ultimately contribute to the reduction in mtDNA content of CKO’s spinal cord.

### Mitochondrial antioxidant capacity is reduced in CKO’s spinal cord

**Figure 2.**
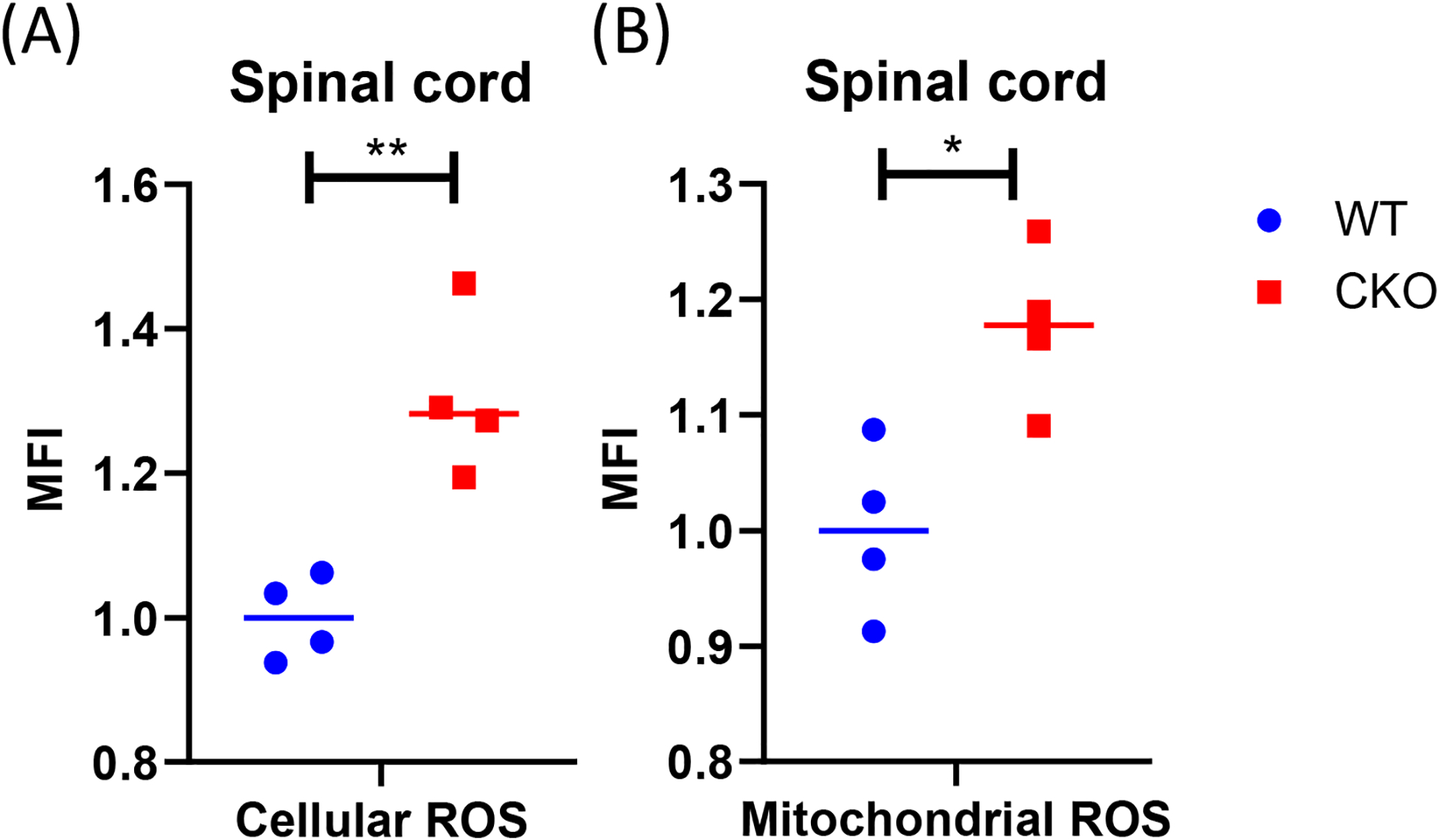
CRABP1 deficiency increases ROS production in spinal cord. Cells from spinal cord tissues were incubated with CellROX (**A**) or MitoSOX **(B)** at 37°C for 30 min and analyzed by flow cytometry. Cells were gated based on single and live cells (propidium iodide^−^). Data were normalized to WT. n=4, pooled results were from two independent experiments. Student’s t-test, *p<0.05, **p<0.01, *** p<0.001 mean fluorescence intensity. MFI: Mean Fluorescence Intensity.

The production of ROS can trigger mtDNA mutations [30,31]. Neurodegenerative disorders, such as ALS, are known to be associated with elevated levels of ROS [32]. Mitochondria generate most of the intracellular ROS, and a persistently elevated ROS level can lead to accumulated DNA, protein, and lipid damage in the cells. All these ultimately affect cellular health and function. Interestingly, in CKO’s spinal cord, both cellular ROS (Figure 2A) and mitochondrial ROS (Figure 2B) levels were elevated, as compared to WT tissues. It is known that ROS is typically generated from normal cellular metabolic processes, mainly in the electron transport chain. Since mitochondrial functions such as respiration in CKO was in fact suppressed (Figure 1D), the increase in ROS levels in CKO could not be caused by enhanced metabolism. Rather, depleting CRABP1 likely caused stress that increased ROS production and damaged CKO cells.

**Figure 3.**
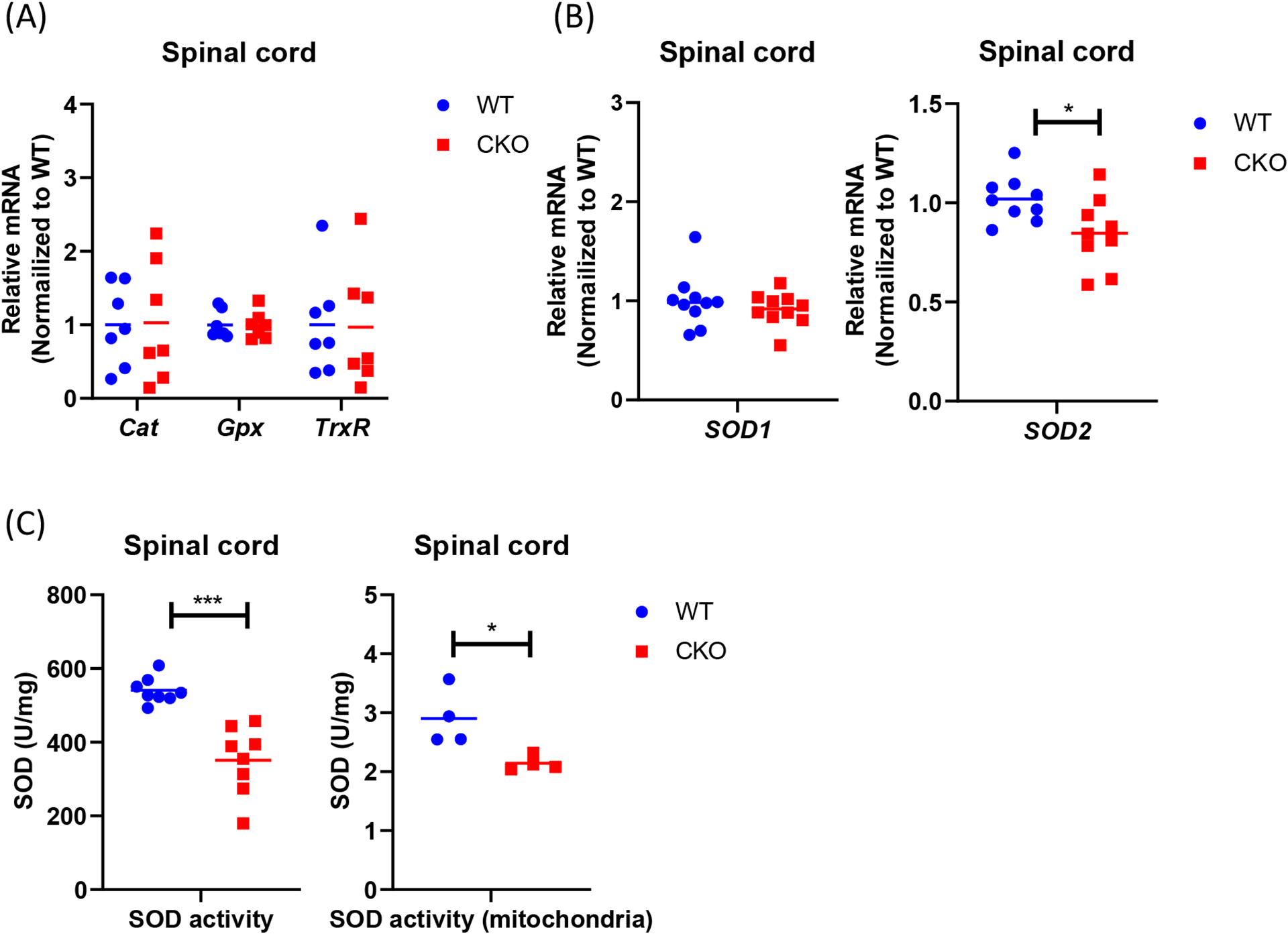
Antioxidant pathway is impaired in CKO spinal cord. **(A)** qPCR to determine the expression of ROS scavenger genes in spinal cord tissues. *RPL19* served as an internal control, and data were normalized to WT. n=7. **(B)** qPCR to determine *SOD* expression in spinal cord tissues. *RPL19* served as an internal control, and data were normalized to WT. n=9–10. **(C)** SOD activities were detected in spinal cord tissues by colorimetric. Total cellular activity, n=7, mitochondrial activity, n=4. Student’s t-test, *p<0.05, **p<0.01, *** p<0.001.

It is known that despite ROS being produced during metabolism, the steady-state level of superoxide is normally guarded by several mechanisms that can facilitate ROS elimination. Therefore, we compared the expression of various antioxidant genes between CKO and WT mice, including *SOD1, SOD2, Cat, Gpx*, and *TrxR1*. Interestingly, a significant reduction in *SOD2* level was detected in CKO’s spinal cord as compared to WT (Figures 3A and 3B). Consistently, both total cellular and mitochondrial SOD activities were lower in CKO’s spinal cord (Figure 3C). Together, these results demonstrate that, in CKO spinal cord, the reduction in *SOD2* leads to decreased SOD activity, especially mitochondrial SOD activity, which may contribute to higher ROS levels, as well as mitochondrial malfunction in CKO tissues.

### UPR^mt^ is impaired in the spinal cord of CKO mice

**Figure 4.**
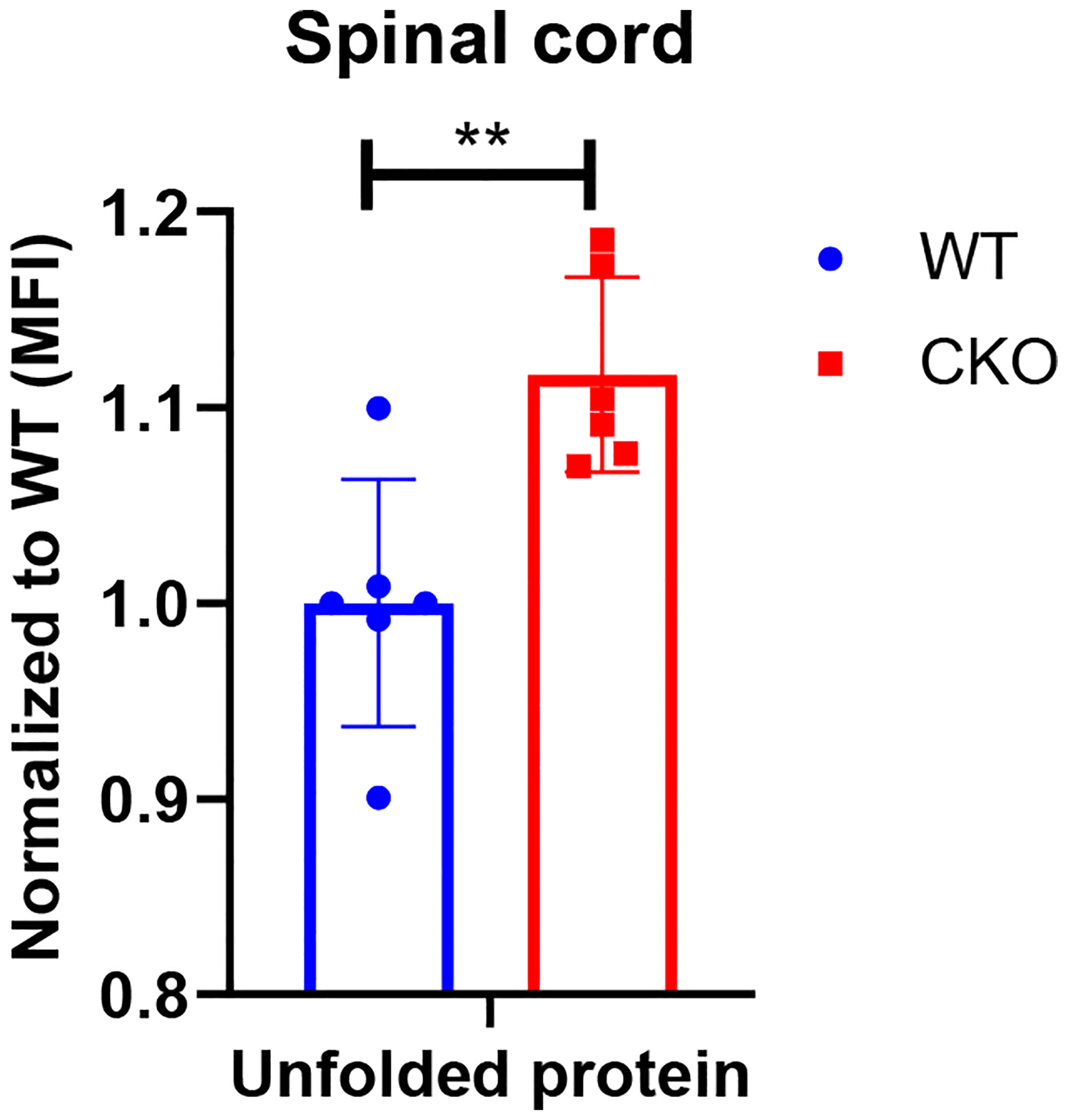
CRABP1 can modulate unfolded protein load in spinal cord. Cells from spinal cord tissues were incubated with 50 μM TPE-MI at 37°C for 30 min and analyzed by flow cytometry. Cells were gated based on single and live cells (propidium iodide^−^). Data were normalized to WT. n=6, pooled results were from three independent experiments. Error bars show means ± SD. Student’s t-test, **p<0.01. MFI = Mean fluorescence intensity.

**Figure 5.**
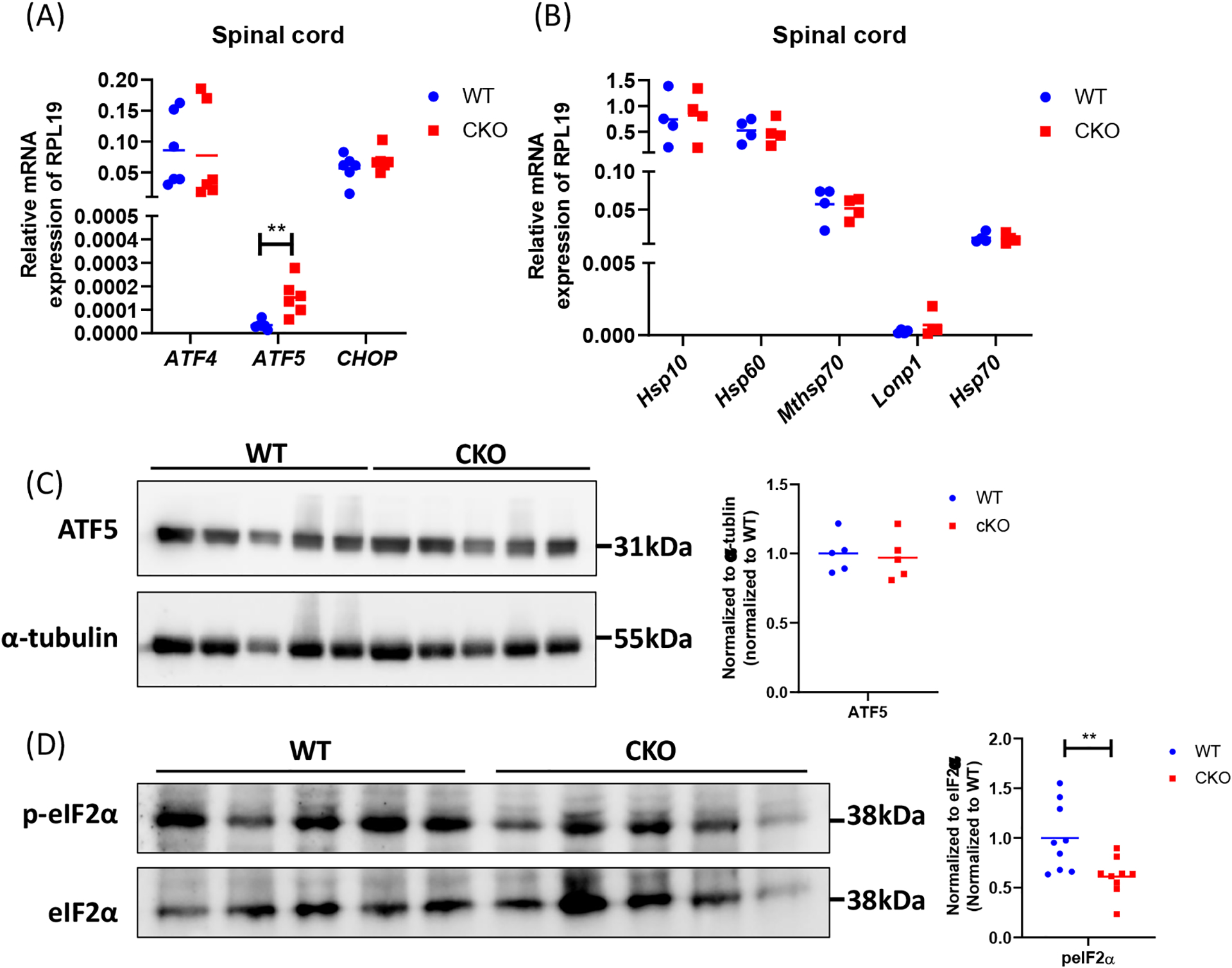
CRABP1 deficiency impairs UPR^mt^ in spinal cord. **(A)** qPCR to determine the expression of UPR^mt^ transcription factors in spinal cord tissues. *RPL19* served as an internal control. n=6. **(B)** qPCR to determine the expression UPR^mt^ genes in spinal cord tissues. *RPL19* served as an internal control. n=6. **(C)** Left: Western blots to examine ATF5 protein expression in spinal cord tissues of WT and CKO mice. Right: Western blot quantification. Total α-tubulin was used as the loading controls. n=5. Data were normalized to WT. **(D)** Left: Western blots of spinal cord tissues harvested from WT and CKO mice to detect the levels of p-eIF2α. Right: quantification of peIF2α levels. Total eIF2α levels served as the loading controls. Data were normalized to WT. Results were obtained from two independent blots. n=9. Student’s t-test, **p<0.01

Excessive production of ROS can damage mitochondrial proteins and DNA, and disrupt mitochondrial function. In normal conditions, mitochondrial unfolded protein response (UPR^mt^) provides an important mitochondrial defense mechanism; thus, defects in UPR^mt^ generally lead to cellular dysfunction/death. To determine if UPR^mt^ was altered in CKO spinal tissues, we first measured the unfolded protein load in spinal cord tissues using a thiol probe, tetraphenylethene maleimide (TPE-MI). The data indeed revealed a significantly higher unfolded protein load in CKO tissues as compared to WT tissues (Figure 4). UPR^mt^ employs, mainly, nuclear translocation of elevated transcription factors *ATF4, ATF5*, and *CHOP* which enhance the expression of key genes that can relieve UPR. It appeared that among *ATF4*, *ATF5*, and *CHOP, ATF5* mRNA level was significantly elevated in CKO tissues (Figure 5A), supporting an increased UPR^mt^ load in CKO’s spinal cord. Surprisingly, the expression of their target genes, such as genes for mitochondrial chaperone proteins and proteases, was not significantly altered (Figure 5B), suggesting that transcriptional enhancement of UPR^mt^ target gene expression was blocked. It appeared that, in spite of the apparent increase in ATF5 mRNA level, ATF5 protein level was not altered in CKO (Figure 5C), which explained the lack of response in the expression of UPR^mt^ genes in CKO (Figure 5B). Furthermore, there was a modest, but statistically significant, decrease in phosphorylated eIF2α (p-eIF2α) level (Figure 5D). Since p-eIF2α is crucial for ATF5 translation [33], a decrease in p-eIF2α would block the expected increase in ATF5 protein level that is required for enhanced transcription of UPR^mt^ genes. Thus, in CKO, while ATF5 mRNA was increased (indicating a stressed state), ATF5 protein level was not elevated; therefore, the execution of UPR^mt^ could not be completed. The failure in ATF5 translational response could be caused by impaired eIF2α phosphorylation. All together, these data show that depleting CRABP1 leads to defected eIF2α phosphorylation, which impairs ATF5 translation that is required for the execution of UPR^mt^ in order to resolve stresses.

### CRABP1 modulates UPR^mt^ in MNs

**Figure 6.**
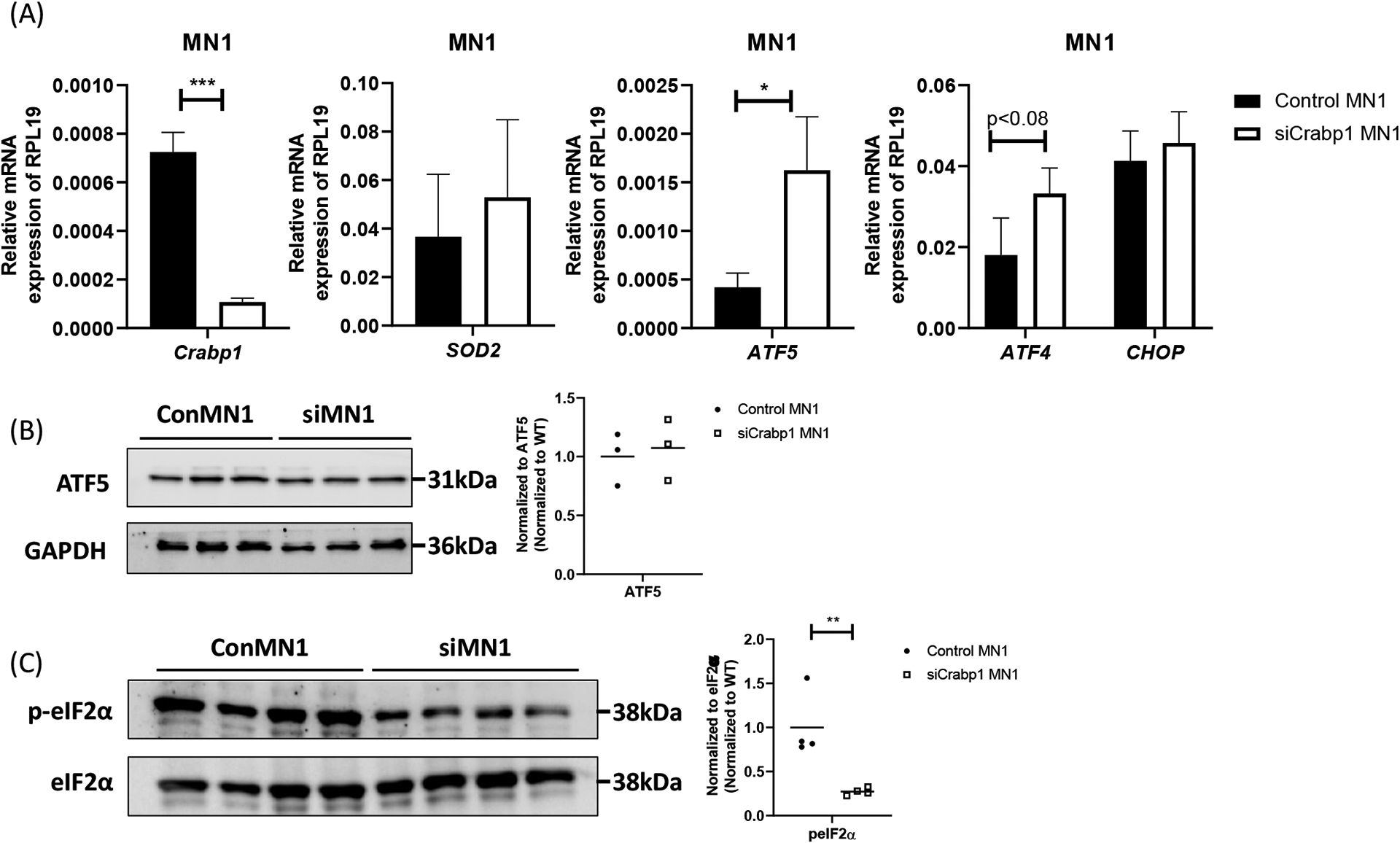
Cell-autonomous function of CRABP1 in modulating UPR^mt^ in MNs. **(A)** qPCR to determine the expression of *Crabp1*, *ATF5, ATF4, CHOP*, *and SOD2* in WT and siCRABP1-MN1 cells. *RPL19* served as an internal control. Error bars show means ± SD. **(B)** Left: Western blots to determine ATF5 protein levels. Right: quantification of ATF4 levels with total GAPDH as loading controls. Data were normalized to WT. **(C)** Left: Western blots to determine p-eIF2α protein levels. Right: quantification of peIF2α levels, with total eIF2α serving as loading controls. Data were normalized to WT. Results were obtained from two independent experiments.

In spinal cord, CRABP1 is specifically and highly expressed only in spinal MNs [7], suggesting the defect in CKO tissues is likely due to the loss of CRABP1 in MNs. To validate if the detected mitochondrial damage in CKO spinal cord is caused by cell-autonomous function of CRABP1 in MNs, we employed a MN cell line, MN1, as an experimental system for further studies. MN1 expresses CRABP1 and displays traits characteristic of MNs [34], thus it is an appropriate model for this study. We generated a CRABP1 knockdown MN cell line, siCrabp1-MN1. We assessed whether this deficiency alone in MNs could affect their UPR^mt^ program. Figure 6A shows that silencing *Crabp1* in MN1 cells readily increased the expression of ATF5 mRNA. The expression of ATF4 mRNA in siCrabp1-MN1 cells was slightly higher than that of control-MN1 cells, which appeared to be different from the data of primary CKO tissue (Figure 5A) (see discussion). Importantly, in siCrabp1-MN1, while ATF5 mRNA level was elevated, its protein level was not increased (Figure 6B). Furthermore, p-eIF2α level was lower in siCrabp1-MN1 cells (Figure 6C). Interestingly, SOD2 level was not altered in siCrabp1-MN1 (Figure 5A), although it was reduced in CKO spinal cord (Figure 3B), suggesting a potential compensatory response of SOD2 in CKO animals. Nevertheless, reduced eIF2α phosphorylation and failure in ATF5 protein increase in siCrabp1-MN1 are consistent with the data of primary CKO mouse tissues, confirming a cell-autonomous function of CRABP1 in modulating eIF2α phosphorylation and the subsequent translational response of ATF5 to alter ATF5 protein level.

## Discussion

**Figure 7.**
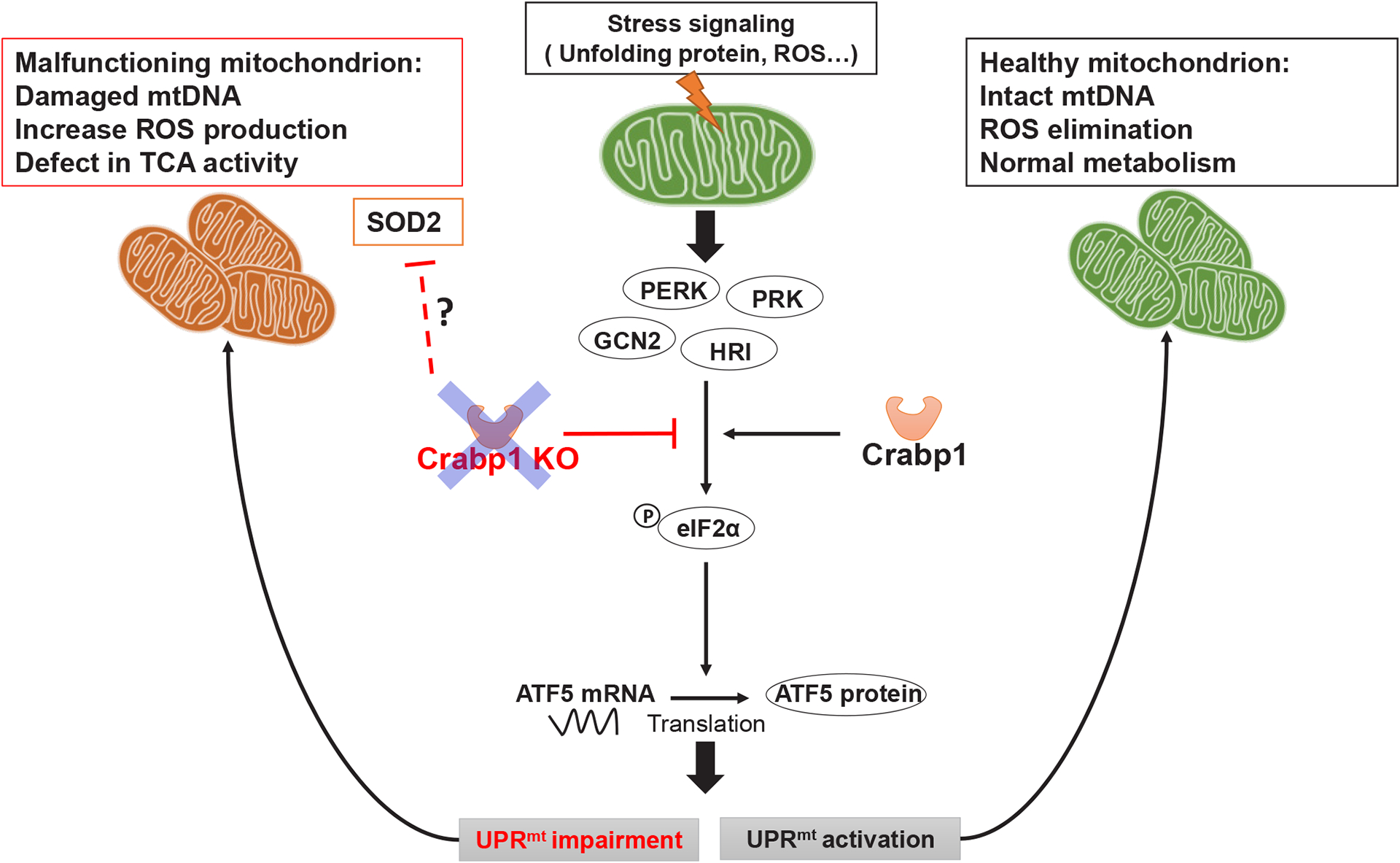
A model for CRABP1’s functional role in maintaining mitochondrial homeostasis. CRABP1 plays a role in maintaining mitochondrial homeostasis by regulating eIF2α phosphorylation for subsequent translational regulation of ATF5, thereby modulating the execution of UPR^mt^. The effect on antioxidant pathway, such as SOD2, in CKO mice could be caused by physiological compensation in whole animals.

In a healthy normal cell, when mitochondria encounter stressors, such as the accumulation of unfolded proteins or oxidative stress, eIF2α is phosphorylated by eIF2α kinases. The phosphorylated eIF2α causes global repression of protein synthesis while specifically increasing the translation of CHOP, ATF4, and ATF5 which are transcription factors for genes associated with the UPR^mt^ [34], thereby mitigating stress. Additionally, the antioxidant pathway is activated, resulting in the upregulation of enzymatic and non-enzymatic antioxidants that help to eliminate excessive ROS accumulation [35]. In this context, this study reveals a new cell-autonomous function of CRABP1 specifically in promoting eIF2α phosphorylation to increase ATF5 protein that enhances transcription of UPR^mt^ target genes. As such, CRABP1 contributes to the maintenance of mitochondrial homeostasis by regulating the execution of UPR^mt^ and, possibly, the antioxidant pathway. Without CRABP1, mitochondrial stresses can accumulate in spinal cord tissue, including accumulation of unfolded proteins, elevated ROS and reduced mitochondrial content. Consequently, a detrimental cycle arises, leading to dysfunctional mitochondria and subsequent MNs dysfunction (model in Figure 7), ultimately damaged motor control. As such, CKO mice develop a progressive ALS-like phenotype characterized by the accumulation of defected NMJs and reduced functional motor units.

eIF2α can be phosphorylated by various eIF2α kinases including general control nonderepressible 2 (GCN2), PKR-like endoplasmic reticulum kinase (PERK), double-stranded RNA-dependent protein kinase (PKR), and heme-regulated inhibitor (HRI). The phosphorylation state of eIF2α can also be regulated by type 1 protein phosphatase complex (PP1c] [36,37]. To investigate whether CRABP1 directly impacts on the activity of eIF2α kinases or PP1c, we performed co-immunoprecipitation experiments to test if CRABP1 physically associated with key components such as eIF2α, PP1c, PERK, and GCN2 in spinal cord tissue. These experiments (unpublished) suggested that endogenous CRABP1 does not physically associate with PERK, GCN2, or PP1c. Further research is needed to carefully determine how CRABP1 affects other factors modulating its activation such as through its upstream signaling, or other transcriptional or post-transcriptional events. Post-translational modifications can regulate protein activity and stability. For instance, ATF5 can undergo ubiquitination, triggered by CDC34; and ATF5 degradation can be suppressed by cadmium and NLK phosphorylation, resulting in stabilization of ATF5 protein. ATF5 can also be degraded through proteasome pathways specifically in hepatocellular carcinoma cells; several miRNAs, such as miR-141–3p, miR-520b-3p, and miR-134–5p, have been identified to bind to the 3’ UTR site of ATF5 mRNA and suppress ATF5 expression [38]. Future studies are needed to determine whether CRABP1 affects ATF5 protein stability.

Currently, RA is the best-known endogenous ligand of CRABP1. The regulation of RA concentration in a physiological context is tightly controlled by various enzymes and metabolic pathways [39–41]. CRABP1 has been proposed to be involved in RA metabolism; presumably, deleting *Crabp1* gene can affect endogenous RA availability (see later). This direction deserves further investigation. However, *in vitro* studies showed apo-CRABP1 readily functional, i.e., purified CRABP1 protein could affect signaling proteins that it can directly interact with, i.e. Rapidly Accelerated Fibrosarcoma 1 (Raf1, a component upstream of ERK signaling pathway) [42], and CaMKII [43]. These interactions consistently resulted in dampened enzymatic activation; therefore, the absence of CRABP1 would cause over-activation of ERK and CaMKII, with or without RA *in vitro*. However, if and how vitamin A status affects these events in a physiological context remains to be investigated.

Additionally, others’ studies have shown that RA can contribute to the regulation of mitochondrial gene transcription and affects mitochondrial number and oxidative phosphorylation [44,45]. It is known that cytochrome P450 (CYP) can maintain tissue RA concentration [46], and CRABP1 could participate in RA metabolism by delivering RA to CYP metabolic enzymes [47,48]. Therefore, it is also a possibility that dysregulated RA concentration, potentially due to the loss of CRABP1, may contribute to defects in eIF2α activation and mitochondrial function by altering endogenous RA availability. Further investigation is needed to determine to what extent CRABP1 may possibly contribute to the maintenance of UPR^mt^ for MN health via its action in modulating intracellular RA content, or by its action in modulating stress execution in MNs as shown in this current study.

The reduction in mtDNA content in CKO’s spinal cord can be attributed to various factors, encompassing interactions with transcription-related proteins, such as mitochondrial transcription factor A (TFAM), potential mitochondrial fusion/fission defects, increased levels of reactive oxygen species (ROS), or decreased efficiency in mtDNA repair mechanisms [49]. Our results have ruled out both fusion/fission-related genes and TFAM level (all remained unchanged between the WT and CKO spinal cord) (Figure 1B). However, we found elevated ROS levels in CKO (Figure 2). It is known that ROS can induce mtDNA mutations [30,31] and is intimately related to stress. Given that eIF2α, a critical player in the translation of transcription factors for genes associated with the UPR^mt^ [34] to mitigate stress, our finding of altered eIF2α activation in CKO (Figure 5) sheds light on a potential direction for further studies of how eIF2α activation is modulated by CRABP1.

Interestingly, in siCrabp1-MN1 model, not only ATF5 mRNA was increased but also ATF4 mRNA slightly increased. However, in CKO spinal cord tissues, only ATF5 was significantly altered. This could be caused by physiological compensation in animals, since in spinal cord, *Crabp1* is highly expressed only in spinal MNs [7]. With regards to various SOD genes, SOD2 was altered in CKO spinal cord, but not in siCRABP1-MN1 cells; this could also be caused by certain physiological compensation. SOD2 expression is highly relevant to neurodegenerative diseases. For instance, in patients of riboflavin transporter deficiency (RTD), a neurodegenerative disorder characterized by sensorineural deafness and motor neuron degeneration, MNs have lowered SOD2 expression [50]. Furthermore, overexpression of SOD2 can attenuate cytotoxicity in mutant SOD1-mediated ALS models [51]. These phenomena are all consistent with the significant reduction in SOD2, and elevation in ROS levels in CKO mice. Further studies are needed to understand whether and how CRABP1 is directly involved in the regulation of SOD2.

Finally, autophagy is a crucial process that maintains cell homeostasis by degrading damaged organelles. Mitophagy process selectively removes damaged or excessive mitochondria, which is typically triggered by damaged mitochondria that lose their membrane potential. Given that MMP was similar between WT and CKO’s spinal cord tissues, mitophagy is most likely not affected in CKO’s spinal cord. However, autophagy can be rapidly activated in response to ROS or aggregates of misfolded proteins [52,53]. Since ROS levels and unfolded protein load were both elevated in CKO spinal tissues, whether autophagy is affected in CKO would present another interesting direction to investigate in the future.

In summary, this study reports a new functional role for CRABP1 in maintaining mitochondrial homeostasis in spinal tissue, especially spinal MNs, by regulating eIF2α phosphorylation and subsequent ATF5 translation, ensuring complete execution of UPR^mt^ in order to resolve stresses. This provides a potential, new mechanistic insight into the spontaneously developed ALS-like motor degenerative phenotype of CKO mice, which gradually lose functional spinal MNs where CRABP1 is otherwise highly expressed to protect cells from stresses that can induce mitochondrial damage.

## Supplementary Material

JCS-23-102_Supplementary File **Table S1:** List of primers for quantitative RT-PCR.
**Figure S1.** CRABP1 deficiency impairs the expression of mtDNA-encoded protein.

## Data Availability

The data generated during this study are available upon reasonable request from the corresponding author.

## References

[1] Wei LN, Chang L, Hu X. Studies of the type I cellular retinoic acid-binding protein mutants and their biological activities. Mol Cell Biochem. 1999;200(1–2):69–76. 10569185 10.1023/a:1006906415388

[2] Nagpal I, Wei LN. All-trans Retinoic Acid as a Versatile Cytosolic Signal Modulator Mediated by CRABP1. Int J Mol Sci. 2019;20(15):3610. 31344789 10.3390/ijms20153610 PMC6696438

[3] Huang P, Chandra V, Rastinejad F. Retinoic acid actions through mammalian nuclear receptors. Chem Rev. 2014;114(1):233–54. 24308533 10.1021/cr400161b PMC3931982

[4] Mey J. RAR/RXR-Mediated Signaling. In: Mandal SS. Gene Regulation, Epigenetics and Hormone Signaling. 2017. p. 457–510.

[5] Nhieu J, Lin YL, Wei LN. CRABP1 in Non-Canonical Activities of Retinoic Acid in Health and Diseases. Nutrients. 2022;14(7):1528. 35406141 10.3390/nu14071528 PMC9003107

[6] Lin YL, Lin YW, Nhieu J, Zhang X, Wei LN. Sonic Hedgehog-Gli1 Signaling and Cellular Retinoic Acid Binding Protein 1 Gene Regulation in Motor Neuron Differentiation and Diseases. Int J Mol Sci. 2020;21(11):4125. 32527063 10.3390/ijms21114125 PMC7312406

[7] Lin YL, Nhieu J, Liu PY, Le G, Lee DJ, Wei CW, CRABP1-CaMKII-Agrn regulates the maintenance of neuromuscular junction in spinal motor neuron. Cell Death Differ. 2022;29(9):1744–56. 35217789 10.1038/s41418-022-00959-4 PMC9433400

[8] Lin YL, Wei CW, Lerdall TA, Nhieu J, Wei LN. Crabp1 Modulates HPA Axis Homeostasis and Anxiety-like Behaviors by Altering FKBP5 Expression. Int J Mol Sci. 2021;22(22):12240. 34830120 10.3390/ijms222212240 PMC8619219

[9] Najjar F, Nhieu J, Wei C-W, Milbauer L, Burmeister L, Seelig D, Deleting Cellular Retinoic-Acid-Binding Protein-1 (Crabp1) Gene Causes Adult-Onset Primary Hypothyroidism in Mice. Endocrines. 2023;4(1):138–50.

[10] Lin YW, Nhieu J, Wei CW, Lin YL, Kagechika H, Wei LN. Regulation of exosome secretion by cellular retinoic acid binding protein 1 contributes to systemic anti-inflammation. Cell Commun Signal. 2021;19(1):69. 34193153 10.1186/s12964-021-00751-w PMC8247179

[11] Wei CW, Nhieu J, Lin YL, Wei LN. Modulation of adipose inflammation by cellular retinoic acid-binding protein 1. Int J Obes (Lond). 2022;46(10):1759–69. 35794192 10.1038/s41366-022-01175-3 PMC9492549

[12] Lin YW, Park SW, Lin YL, Burton FH, Wei LN. Cellular retinoic acid binding protein 1 protects mice from high-fat diet-induced obesity by decreasing adipocyte hypertrophy. Int J Obes (Lond). 2020;44(2):466–74. 31164723 10.1038/s41366-019-0379-z PMC6891142

[13] Maeda M, Harris AW, Kingham BF, Lumpkin CJ, Opdenaker LM, McCahan SM, Transcriptome profiling of spinal muscular atrophy motor neurons derived from mouse embryonic stem cells. PLoS One. 2014;9(9):e106818. 25191843 10.1371/journal.pone.0106818 PMC4156416

[14] Rizzo F, Nizzardo M, Vashisht S, Molteni E, Melzi V, Taiana M, Key role of SMN/SYNCRIP and RNA-Motif 7 in spinal muscular atrophy: RNA-Seq and motif analysis of human motor neurons. Brain. 2019;142(2):276–94. 30649277 10.1093/brain/awy330 PMC6351774

[15] Swindell WR, Bojanowski K, Kindy MS, Chau RMW, Ko D. GM604 regulates developmental neurogenesis pathways and the expression of genes associated with amyotrophic lateral sclerosis. Transl Neurodegener. 2018;7:30. 30524706 10.1186/s40035-018-0135-7 PMC6276193

[16] Zhunina OA, Yabbarov NG, Grechko AV, Starodubova AV, Ivanova E, Nikiforov NG, The Role of Mitochondrial Dysfunction in Vascular Disease, Tumorigenesis, and Diabetes. Front Mol Biosci. 2021;8:671908. 34026846 10.3389/fmolb.2021.671908 PMC8138126

[17] Johri A, Beal MF. Mitochondrial dysfunction in neurodegenerative diseases. J Pharmacol Exp Ther. 2012;342(3):619–30. 22700435 10.1124/jpet.112.192138 PMC3422529

[18] Smith EF, Shaw PJ, De Vos KJ. The role of mitochondria in amyotrophic lateral sclerosis. Neurosci Lett. 2019;710:132933. 28669745 10.1016/j.neulet.2017.06.052

[19] Birben E, Sahiner UM, Sackesen C, Erzurum S, Kalayci O. Oxidative stress and antioxidant defense. World Allergy Organ J. 2012;5(1):9–19. 23268465 10.1097/WOX.0b013e3182439613 PMC3488923

[20] Suarez-Rivero JM, Pastor-Maldonado CJ, Povea-Cabello S, Alvarez-Cordoba M, Villalon-Garcia I, Talaveron-Rey M, Activation of the Mitochondrial Unfolded Protein Response: A New Therapeutic Target? Biomedicines. 2022;10(7):1611. 35884915 10.3390/biomedicines10071611 PMC9313171

[21] Fox TD. Mitochondrial protein synthesis, import, and assembly. Genetics. 2012;192(4):1203–34. 23212899 10.1534/genetics.112.141267 PMC3512135

[22] Slavin MB, Kumari R, Hood DA. ATF5 is a regulator of exercise-induced mitochondrial quality control in skeletal muscle. Mol Metab. 2022;66:101623. 36332794 10.1016/j.molmet.2022.101623 PMC9661517

[23] Kasai S, Yamazaki H, Tanji K, Engler MJ, Matsumiya T, Itoh K. Role of the ISR-ATF4 pathway and its cross talk with Nrf2 in mitochondrial quality control. J Clin Biochem Nutr. 2019;64(1):1–12. 30705506 10.3164/jcbn.18-37 PMC6348405

[24] Melber A, Haynes CM. UPR(mt) regulation and output: a stress response mediated by mitochondrial-nuclear communication. Cell Res. 2018;28(3):281–95. 29424373 10.1038/cr.2018.16 PMC5835775

[25] Gorry P, Lufkin T, Dierich A, Rochette-Egly C, Decimo D, Dolle P, The cellular retinoic acid binding protein I is dispensable. Proc Natl Acad Sci U S A. 1994;91(19):9032–6. 8090764 10.1073/pnas.91.19.9032 PMC44741

[26] Pino PA, Cardona AE. Isolation of brain and spinal cord mononuclear cells using percoll gradients. J Vis Exp. 2011(48):2348. 21339713 10.3791/2348 PMC3339837

[27] Wang W, Qi B, Lv H, Wu F, Liu L, Wang W, A new method of isolating spinal motor neurons from fetal mouse. J Neurosci Methods. 2017;288:57–61. 28648716 10.1016/j.jneumeth.2017.06.014

[28] Piantadosi CA, Suliman HB. Mitochondrial transcription factor A induction by redox activation of nuclear respiratory factor 1. J Biol Chem. 2006;281(1):324–33. 16230352 10.1074/jbc.M508805200

[29] Chen MZ, Moily NS, Bridgford JL, Wood RJ, Radwan M, Smith TA, A thiol probe for measuring unfolded protein load and proteostasis in cells. Nat Commun. 2017;8(1):474. 28883394 10.1038/s41467-017-00203-5 PMC5589734

[30] Nissanka N, Moraes CT. Mitochondrial DNA damage and reactive oxygen species in neurodegenerative disease. FEBS Lett. 2018;592(5):728–42. 29281123 10.1002/1873-3468.12956 PMC6942696

[31] Hahn A, Zuryn S. Mitochondrial Genome (mtDNA) Mutations that Generate Reactive Oxygen Species. Antioxidants (Basel). 2019;8(9):392. 31514455 10.3390/antiox8090392 PMC6769445

[32] Barber SC, Shaw PJ. Oxidative stress in ALS: key role in motor neuron injury and therapeutic target. Free Radic Biol Med. 2010;48(5):629–41. 19969067 10.1016/j.freeradbiomed.2009.11.018

[33] Zhou D, Palam LR, Jiang L, Narasimhan J, Staschke KA, Wek RC. Phosphorylation of eIF2 directs ATF5 translational control in response to diverse stress conditions. J Biol Chem. 2008;283(11):7064–73. 18195013 10.1074/jbc.M708530200

[34] Shpilka T, Haynes CM. The mitochondrial UPR: mechanisms, physiological functions and implications in ageing. Nat Rev Mol Cell Biol. 2018;19(2):109–20. 29165426 10.1038/nrm.2017.110

[35] Sindhu RK, Kaur P, Kaur P, Singh H, Batiha GE, Verma I. Exploring multifunctional antioxidants as potential agents for management of neurological disorders. Environ Sci Pollut Res Int. 2022;29(17):24458–77. 35064486 10.1007/s11356-021-17667-0

[36] Wek RC. Role of eIF2alpha Kinases in Translational Control and Adaptation to Cellular Stress. Cold Spring Harb Perspect Biol. 2018;10(7):a032870. 29440070 10.1101/cshperspect.a032870 PMC6028073

[37] Choy MS, Yusoff P, Lee IC, Newton JC, Goh CW, Page R, Structural and Functional Analysis of the GADD34:PP1 eIF2alpha Phosphatase. Cell Rep. 2015;11(12):1885–91. 26095357 10.1016/j.celrep.2015.05.043 PMC4489983

[38] Paerhati P, Liu J, Jin Z, Jakos T, Zhu S, Qian L, Advancements in Activating Transcription Factor 5 Function in Regulating Cell Stress and Survival. Int J Mol Sci. 2022;23(13):7129. 35806136 10.3390/ijms23137129 PMC9266924

[39] Kedishvili NY. Enzymology of retinoic acid biosynthesis and degradation. J Lipid Res. 2013;54(7):1744–60. 23630397 10.1194/jlr.R037028 PMC3679379

[40] Han BC, Xia HF, Sun J, Yang Y, Peng JP. Retinoic acid-metabolizing enzyme cytochrome P450 26a1 (cyp26a1) is essential for implantation: functional study of its role in early pregnancy. J Cell Physiol. 2010;223(2):471–9. 20112286 10.1002/jcp.22056

[41] Blaner WS. Vitamin A signaling and homeostasis in obesity, diabetes, and metabolic disorders. Pharmacol Ther. 2019;197:153–78. 30703416 10.1016/j.pharmthera.2019.01.006 PMC6520171

[42] Park SW, Nhieu J, Persaud SD, Miller MC, Xia Y, Lin YW, Publisher Correction: A new regulatory mechanism for Raf kinase activation, retinoic acid-bound Crabp1. Sci Rep. 2019;9(1):17042. 31728066 10.1038/s41598-019-53692-3 PMC6856344

[43] Park SW, Persaud SD, Ogokeh S, Meyers TA, Townsend D, Wei LN. CRABP1 protects the heart from isoproterenol-induced acute and chronic remodeling. J Endocrinol. 2018;236(3):151–65. 29371236 10.1530/JOE-17-0613 PMC5815894

[44] Tourniaire F, Musinovic H, Gouranton E, Astier J, Marcotorchino J, Arreguin A, All-trans retinoic acid induces oxidative phosphorylation and mitochondria biogenesis in adipocytes. Journal of Lipid Research. 2015;56(6):1100–9. 25914170 10.1194/jlr.M053652 PMC4442868

[45] Everts HB, Berdanier CD. Regulation of mitochondrial gene expression by retinoids. IUBMB Life. 2002;54(2):45–9. 12440518 10.1080/15216540214316

[46] Ross AC, Zolfaghari R. Cytochrome P450s in the regulation of cellular retinoic acid metabolism. Annu Rev Nutr. 2011;31:65–87. 21529158 10.1146/annurev-nutr-072610-145127 PMC3789243

[47] Napoli JL. Cellular retinoid binding-proteins, CRBP, CRABP, FABP5: Effects on retinoid metabolism, function and related diseases. Pharmacol Ther. 2017;173:19–33. 28132904 10.1016/j.pharmthera.2017.01.004 PMC5408321

[48] Nelson CH, Peng CC, Lutz JD, Yeung CK, Zelter A, Isoherranen N. Direct protein-protein interactions and substrate channeling between cellular retinoic acid binding proteins and CYP26B1. FEBS Lett. 2016;590(16):2527–35. 27416800 10.1002/1873-3468.12303 PMC4997814

[49] Rong Z, Tu P, Xu P, Sun Y, Yu F, Tu N, The Mitochondrial Response to DNA Damage. Front Cell Dev Biol. 2021;9:669379. 34055802 10.3389/fcell.2021.669379 PMC8149749

[50] Colasuonno F, Bertini E, Tartaglia M, Compagnucci C, Moreno S. Mitochondrial Abnormalities in Induced Pluripotent Stem Cells-Derived Motor Neurons from Patients with Riboflavin Transporter Deficiency. Antioxidants (Basel). 2020;9(12):1252. 33317017 10.3390/antiox9121252 PMC7763948

[51] Zimmerman MC, Oberley LW, Flanagan SW. Mutant SOD1-induced neuronal toxicity is mediated by increased mitochondrial superoxide levels. J Neurochem. 2007;102(3):609–18. 17394531 10.1111/j.1471-4159.2007.04502.x

[52] Fang C, Gu L, Smerin D, Mao S, Xiong X. The Interrelation between Reactive Oxygen Species and Autophagy in Neurological Disorders. Oxid Med Cell Longev. 2017;2017:8495160. 29391926 10.1155/2017/8495160 PMC5748124

[53] Filomeni G, De Zio D, Cecconi F. Oxidative stress and autophagy: the clash between damage and metabolic needs. Cell Death and Differentiation. 2015;22(3):377–88. 25257172 10.1038/cdd.2014.150 PMC4326572

