# Supplementary material for: Depleting Cellular Retinoic Acid Binding Protein 1 Impairs UPRmt
Source: J Cell Signal. Author manuscript; Available in PMC 2024 May 3. (PMC11068023; doi:10.33696/signaling.4.102)
Supplement: JCS-23-102_Supplementary File — Table S1: List of primers for quantitative RT-PCR. Figure S1. CRABP1 deficiency impairs the expression of mtDNA-encoded protein. [file NIHMS1939959-supplement-JCS-23-102_Supplementary_File.pdf]

Table S1. List of primers for quantitative RT-PCR

Gene Name	Primer Sequence (Forward)	Primer Sequence (Reverse)
Pyruvate dehydrogenase complex (Pdhx)	5'-GTATCCATTCCTGGGCAACCGA-3'	5'-TAGAACTGCGCCAAGGTCACAG-3'
Mouse ubiquinol-cytochrome c reductase core protein 1 (Uqcrc1)	5'-AGTGTGGATTGACGCTGGCAGT-3'	5'-CCTCCTTCTCTAAGGCATTGCC-3'
Iisocitrate dehydrogenase 3 (NAD+) alpha (Idh3a)	5'-GCAGGACTGATTGGAGGTCTTG-3'	5' GCCATGTCCTTGCCTGCAATGT-3'
peroxisome proliferative activated receptor, gamma, coactivator 1 alpha (Pgc1a)	5'- GAATCAAGCCACTACAGACACCG-3'	5'- CATCCCTCTTGAGCCTTTCGTG-3'
Mitochondrial sirtuin 3 (Sirt3)	5'-CTACATGCACGGTCTGTCGAA-3'	5'-CAATGTCGGGTTTCACAACGC-3'
Mitochondrial transcription factor A (TFAM)	5'-GCAAAGGATGATTCGGCTC-3'	5'-CGAATCCTATCATCTTTAGCAAGC-3'
Mitofusin-1 (MFn1)	5'- CCA GGT ACA GAT GTC ACC ACA G-3'	5'- TTG GAG AGC CGC TCA TTC ACC T-3'
Mitofusin-2 (MFn2)	5'-GTGGAATACGCCAGTGAGAAG C-3'	5'-CAACTTGCTGGCACAGATGAGC-3'
Mitochondrial fission protein 1 (Fis)	5'-GCTGGTTCTGTGTCCAAGAGCA-3'	5'-GACATAGTCCCGCTGTTCCTCT-3'
Some optic atrophy 1 (Opa1)	5'-TCTCAGCCTTGCTGTGTCAGAC-3'	5'-TTCCGTCTCTAGGTTAAAGCGCG-3'
Dynamin-related protein 1, DNM1L (Drp1)	5'-GCGAACCTTAGAATCTGTGGACC-3'	5'-CAGGCACAAATAAAGCAGGACGG-3'
Superoxide dismutase type 1 (SOD1)	5'-GGTGAACCAGTTGTGTTGTCAG-3'	5'-ATGAGGTCCTGCACTGGTAC-3'
Superoxide dismutase type 2 (SOD2)	5'-TAACGCGCAGATCATGCAGC-3'	5'-GCTGAAGAGCGACCTGAGTT-3'
Catalase (Cat)	5'-CAAGATTGCCTTCTCCGGGT-3'	5'-GACATCAGGTCTCTGCGAGG -3'
Thioredoxin Reductase 1, Txnrd1 (TrxR1)	5'-AGTCACATCGGCTCGCTGAA -3'	5'-GATGAGGAACCGCTCTGCTG -3'
Glutathione peroxidase (gpx1)	5'-GCTCTTTACCTTCCTGCGGA-3'	5'-GTTCCAGGCAATGTCGTTGC-3'
Heat Shock Protein Family A Member, Hspa1a (Hsp70)	5'-GGCTGGTGAGCCACTTCGT-3'	5'-GTTCTGGCTGATGTCCTTCTTGT-3'
Stress-70 protein, mitochondrial, HSPA9 (mtHsp70)	5'-CTCTGGGAGGCGTCTTTACC-3'	5'-CGTTCCCCCTGACACACTTT-3'
Heat shock 10kDa protein 1, Hspe1 (Hsp10)	5'-CTGCCGAAACTGTAACCAAAG-3'	5'-TCTCCAACTTTCACACTGACAG-3'
LON peptidase 1 (Lonp1)	5'-TCCGACTTGCACAGCCCTAT-3'	5'-CGTCCGACTCATTGTTGTCATC-3'
Heat Shock Protein Family D Member 1, Hspd1 (Hsp60)	5'-CTGGGTGCAAGAGCCATATA-3'	5'-GAAAGGCTGCTTCTGAACTCT-3'
Activating transcription factor 4 (ATF4)	5'-GCCGGTTTAAGTTGTGTGCT-3'	5'-CTGGATTCGAGGAATGTGCT-3'
Activating transcription factor 5 (ATF5)	5'-GGCTGGCTCGTAGACTATGG-3'	5'-CCAGAGGAAGGAGAGCTGTG-3'
DNA damage inducible Transcript 3, Ddit3 (CHOP)	5'-CCTAGCTTGGCTGACAGAGG-3'	5'-CTGCTCCTTCTCCTTCATGC-3'
Cellular retinoic acid binding protein 1 (Crabp1)	5’ CGGAGATCAACTTCAAGGTCGG 3’	5’ CCCTCAAGAAGTGTCTGTGTGC 3’
RPL19	5’ TCATCCGCAAGCCTGTGACT 3’	5’ CTTCTCAGGCATCCGAGCAT 3’
GAPDH	5’ ACTCCACTCACGGCAAATTC 3’	5’ TCTCCATGGTGGTGAAGACA 3’.

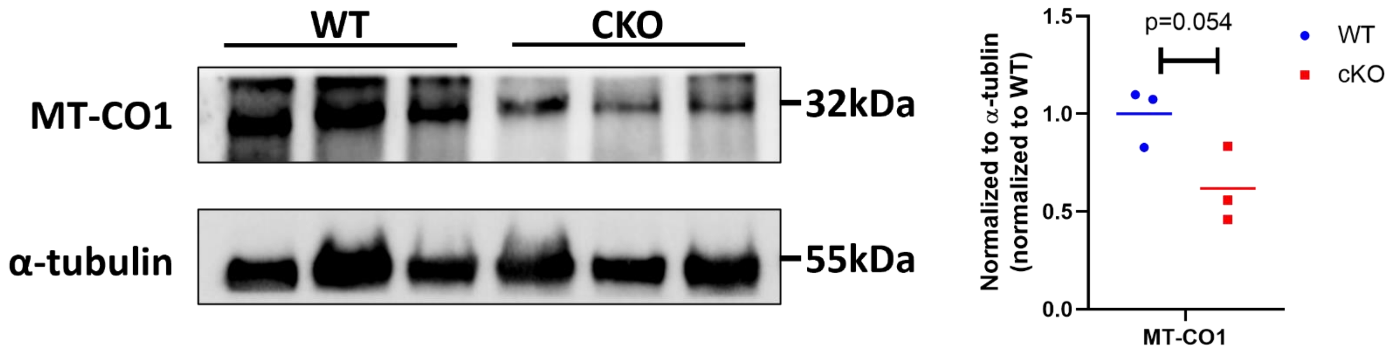

Figure S1. CRABP1 deficiency impairs the expression of mtDNA-encoded protein.

Left: Western blot for comparing the expression of MT-CO1 (#62101, Cell Signaling) in spinal cord tissues of 3-month-old WT and CKO mice. Right: Quantification of Western blot data. Total α-tubulin was used as the loading controls. n=3. Data were normalized to WT.
